# It’s not you (well, it is a bit you), it’s me: Self- versus social image in warm-glow giving

**DOI:** 10.1371/journal.pone.0300868

**Published:** 2024-03-25

**Authors:** Philip J. Grossman, Jonathan Levy

**Affiliations:** 1 Department of Economics, Monash University, Victoria, Australia; 2 School of Economics, The University of Sydney, Camperdown, New South Wales, Australia; Georg-August-Universität Göttingen: Georg-August-Universitat Gottingen, GERMANY

## Abstract

Attempts by charities to motivate giving tend to focus on potential donors’ altruistic tendencies. However, prior research suggests that approximately 50% of individuals are to some extent motivated by warm glow, the satisfaction received from the act of giving. The satisfaction derives from looking good to themselves (self-image) and/or to others (social image). We conduct an online experiment on MTurk participants (*n* = 960) with a more realistic simulation of being watched to determine the importance of self- and social image to warm-glow giving. We find evidence that suggests that social image concerns do not increase the likelihood that someone will give but they do increase the amount given; average giving is significantly higher in the treatments when feelings of being watched are stimulated. Our results suggest that charities looking to increase their donor bases might effectively do so by focusing on self-image concerns. Charities wishing to increase the amount donated might effectively do so by focusing on the social image concerns of the donor.

## 1. Introduction

Both pure altruists and pure egoists might give to a charitable organization; the pure altruists out of concern for the well-being of the recipients, the pure egoists (unconcerned with the welfare of recipients) to receive warm glow from the act of giving [1, 2]. Impure altruists are motivated by both altruism and warm glow. Our focus is on warm-glow motivated giving.

The warm-glow utility received from giving to a charity may result from enhancement of either self- or social image. It has been argued that prosocial behavior (which encompasses warm-glow behavior) may be motivated by social image concerns [3–8], the desire for acclaim and recognition. [9, 10] have suggested prosocial behavior may be motivated by a desire to avoid negative feelings such as shame and guilt and thereby maintain self-respect.

The theory of prosocial behavior in [11] combines both concerns for social image, i.e., identity or face presented to others in public contexts, and self-image, i.e., one’s view or concept of oneself (https://dictionary.apa.org/social-image; https://dictionary.apa.org/self-image). The self-perception theory in [12] argues that individuals “know” themselves partially via internal cues, but that these are “… weak, ambiguous, or uninterpretable [12, p.2].” They must also rely on external cues, like an outside observer, if they are to infer their own inner state; they “know” they are good by the actions they take. Image motivation (i.e., “… the desire to be liked and respected by others and *by one’s self*”) is argued to be motivation for behaving prosocially [13].

If individuals wish to hold a positive image of themselves and have others hold a positive image of them, then they must act in such a way that reinforces the desired image. Doing one’s part helps to create and support the desired image. This does not require that in every instance an individual must donate when asked, just that donations are made frequently enough to be consistent with the desired image.

We report results from an online experiment using participants from the Amazon Mechanical Turk (MTurk) subject pool testing the importance of self- and social image for warm-glow giving. All participants play [14]. Dictator game with a charity as the recipient. Dictators and their chosen charities each receive the same endowment amount. The dictators decide how much of their endowment to pass to their chosen charities, knowing that their giving is crowded out $1:$1. Our concern is with warm-glow motivated giving, which could be driven by self- and social image. The [14] design eliminates altruistic motivations for giving (i.e., knowing that giving creates no benefits for the clients of the charity, a pure altruist would have no incentive to give). According to [14] pure altruists could maximize their own utility and benefits for the recipients by not donating during the experiment, maximizing their earnings, and donating on their own after the experiment.

Participants are randomly assigned to one of three treatments: the standard game (with self-image influences but minimal to no social image influence); the social image influence game in which participants are “watched” by a pair of dynamic eyes as they make their donation decisions; and the social image influence choice game in which participants have the option of turning off the watching eyes before making their donation decisions. For the watching eyes, we use a recording of a pair of actual eyes on a 26-second video loop. All other features of the experiment are the same across treatments.

It is worth noting that we take a light touch (relative to other approaches but a heavier touch relative to studies that have employed nondynamic and stylized images of eyes to introduce the appearance of being watched, discussed below) when introducing social image. We considered other methods for introducing social image concerns. For example, social image concerns could have been introduced by having participants observe one another in the laboratory as they make their donation decisions. We dismissed this approach as it risked introducing influences which are not necessarily related to social image, e.g., attractiveness, gender, reciprocity, and power. Moreover, these stronger manipulations are quite difficult to scale up. Although our method for introducing social image employs a somewhat light touch, it enables us to control for these unintended influences. Furthermore, if successful it offers a simple, low-cost mechanism that charitable organizations could use to influence donations. For example, charitable organizations could include a recording of eyes (or the face of a typical recipient) on their websites where people make their donations.

The results from our experiment suggest that self-image is the primary driver for warm-glow giving, accounting for the majority of giving. Roughly 50% of all participants make a positive donation and the probability of making a positive donation does not significantly vary across treatments. Self-image driven giving averages $0.25 (12.5% of the endowment). Social image driven giving significantly increases by an additional $0.08 on average. The results also imply that once the eyes have been seen, the social image effect remains; even if a participant chooses to turn the eyes off, the effect of the eyes cannot be turned off.

## 2. Literature review

Warm glow is a nebulous term; it has typically been defined as the private, emotional pleasure people receive from “doing their part” to help others [1, 2]. Warm glow has been used to justify a variety of behaviors. For example, people may vote, even though it is unlikely that their vote will be pivotal, because they wish to receive the warm glow obtained from voting and/or being seen to vote [15]. Similarly, individuals may drive environmentally friendly vehicles to signal their concern for the environment. Work by [8, 16, 17] suggests that the utility from warm glow is transitory. In their studies, the demand for warm-glow utility was satiated with the initial decision.

There is considerable evidence that some giving is warm-glow motivated. The first to test the warm-glow hypothesis of [1, 2] was [14]. Approximately 50% of their subjects gave even though their giving was crowded out $1:$1, and donations averaged roughly 20% of subjects’ endowments. Subsequent laboratory and online studies using [14] basic design report similar results [16, 18–20]. Using a different design, [17] report that 41% of their sample were warm-glow givers. While these studies provide evidence of warm glow, they do not address whether warm glow derives from having a positive self-image, a positive social image, or from both. We seek to determine the relative importance of self- and social image for warm-glow giving by varying the feeling of being watched.

Self-image is an explanation for warm-glow giving; participants in the studies mentioned above are obviously aware of their giving decisions. Creating a counterfactual in which self-image is switched off is difficult. Several studies have, however, attempted to increase the salience of self-image. One does so by presenting subjects with real-time webcam feeds of themselves [21]. A second reports results from a natural experiment: Customers buying opera tickets through an online booking agent were asked at the time of checkout if they were willing to donate to a charity supporting disadvantaged children [22]. In one treatment, customers who did not donate had the option of ticking a box indicating they had already donated or a second box saying “No, thank you.” Relative to their baseline, the box ticking significantly increased the probability of giving and the average amount given. A third study increased self-image salience by requiring subjects to wear bracelets to remind them of their donations [23]. To assess the impact of social image, [13, 24] compare private and public choices, attributing any difference to social image concerns. A negative correlation between intrinsic motivation and image concerns when comparing public and private willingness-to-pay for fair trade of conventional chocolate is reported by [25].

It should be noted that these studies do not directly address warm-glow giving; they measure both altruistic as well as warm-glow giving. Our design with the $1:$1 crowding out eliminates altruistic motivations for giving (a pure altruist would have no incentive to give since giving provides no benefits to a charity’s beneficiaries). It offers a cleaner test of the impact of self- and social image on warm-glow giving.

Our paper contributes to several different literatures. One literature of particular relevance explores the impact of the appearance of being watched on prosocial behavior: the use of observable static pictures of eyes, stylized images, and three dots resembling the placement of eyes and nose on a face. The effectiveness of this manipulation is the subject of debate. Studies of the effect on dictators’ behavior or in laboratory experiments have been mixed [26–29]. Meta-analysis of seven studies reports that the static watching eyes significantly increased the probability of passing a positive amount, but not the average amount passed [29]. It is argued that only “watching” eyes increase prosocial behavior [30]. Watching eyes are static pictures of real eyes looking straight ahead rather than up, down, or off to the side. In laboratory and field studies using charities as recipients, the results are more positive [31–33]. More recent meta studies suggest that the impact of static watching eyes is only reliably positive in the reduction of antisocial behavior, not in increasing prosocial behavior [34–36].

Our study is distinct from this prior research in three ways. First, we elicit the sensation of being “watched” in a new and more realistic way. Rather than static images of actual or stylized eyes, we employ a recording of a pair of actual eyes on a video loop. Second, our study directly tests the relative importance of self- and social image for warm-glow giving. The fact that prior research found the influences of eyes are mixed is consistent with our finding suggesting that social image is relatively less important than self-image with respect to warm-glow giving decisions. Third, in one of our treatments, we allow for participants to turn off the image of the eyes and make their giving decision without feeling as though they are being watched. In many instances, individuals can choose to make their decisions while being observed by others or, possibly, delay doing so until no one is watching. These novel methodological features provide a richer, more realistic context for us to examine the relative significance of self- and social image for warm-glow giving.

We also contribute to the “reluctant altruism” literature [37–41]. Reluctant altruists want to believe themselves to be prosocial and to display to others their prosociality, without bearing the cost of being prosocial. Presented with a donation request, they face either the monetary cost of giving or the loss in self- and social image of not acting prosocially. Both self- and social image can be saved by either having reasons (i.e., “moral wiggle room,” [42]) that justifies not giving or by sidestepping the giving requests [43, 44]. When individuals are offered the moral wiggle room of opting out of playing the Dictator game, many take the option and giving is significantly less [45–47] offer contrary evidence from a trust game. Donors use a charity’s high overhead as an excuse not only to reduce giving but also to not give at all [48]. Field experiments report that subjects exert effort (i.e., using a less convenient exit) to avoid being asked [43, 49–51]. In one of our treatments, we allow for participants to “turn off” the watching eyes before they make their donation decisions. A reluctant altruist may wish to turn the eyes off before donating a small amount of money; being “observed” not giving may have a negative impact on social image.

Our third contribution is to the social distance literature. Decisions in [14] style games are not totally anonymous; there is some element of social image at play. For, example, in laboratory experiments, participants’ decisions are often exposed when they complete and sign receipt forms. Social image concerns in online experiments using MTurk should be virtually irrelevant as participants (identified only by ID numbers) are anonymous to the experimenters and no receipt form is signed. Social image concerns arise when social distance is reduced and the anonymity of the individual and the individual’s actions are lessened. Increasing social distance is shown to significantly reduces prosocial behavior [52, 53]. They vary the degree of social distance from complete anonymity (i.e., the double-blind protocol; neither the recipient nor the experimenter can identify the dictator or the dictator’s decision) to a single-blind protocol (i.e., while the recipient cannot identify the dictator, the experimenter can identify the dictator as well as the dictator’s allocation decision). Even splits of the endowment in the Dictator game increase with the decrease in the anonymity of the dictators’ decisions [54]. Relative to a no picture treatment, [55] shows that giving significantly increases when dictators (recipients) receive pictures of their recipients (dictators); however, there is no significant difference across treatments in the likelihood of giving. Our experiment offers additional evidence for the social distance hypothesis whereby the feeling of being watched reduces social distance.

## 3. Methodology

The experiment, consisting of five treatments, was preregistered at the Open Science Framework; only three treatments were conducted (Self- versus social image in warm-glow giving: Date created: 2020-07-02 09:37 AM | Last Updated: 2020-08-03 09:11 AM: https://osf.io/ej7d3/). When we preregistered the experiment, we preregistered treatments we initially thought to be relevant. As we progressed in our preparation, but prior to collecting any data, we concluded that two of our proposed treatments, the “static eyes” and the “pay to turn off the dynamic eyes” treatments, were unlikely to add anything of interest to the study and would only add to the cost. We believed that if the dynamic eyes had no effect, the static eyes would also have no effect and that adding a cost to turn off the dynamic eyes would possibly result in too few eyes-turned-off observations. Consequently, we did not collect data for these two treatments. In hindsight, we should have amended our preregistration. The Monash University Human Research Ethics Committee approved the protocol for this study (Project ID: 25385).

The survey was programmed in Qualtrics and conducted between August and November 2020 with participants registered on MTurk. To improve the quality of data collected, we restricted participation to individuals located in the United States with a high approval rate (80%) in their previously completed Human Intelligence Tasks (HITs) and included comprehension check questions. Participants were identified by their MTurk IDs, no identifying information was collected. This experiment is a between-subject design.

The experiment consists of three parts. In part 1, participants receive instructions related to the warm-glow giving task. Participants select from a list of ten, a charity to receive any donation they make (see S1 Appendix). They then have two tries to correctly answer two comprehension check quiz questions testing their understanding of the task (in particular, that any donation was crowded out $1:$1). Participants answering one or more of the questions incorrectly on both tries were not allowed to complete the remainder of the experiment. After completing their donation decisions, participants completed parts 2 and 3; a demographic survey and a 10-item Big Five Personality traits test taken from [56], respectively. Questions from parts 2 and 3 are in S1 Appendix.

Participants who successfully answered both part 1 quiz questions were randomly assigned to one of three treatments:

*Treatment 1 (***NoEyes***)*: Standard game as in [14]. Both participants and charity of choice receive endowments of 200 ECU where 1 ECU = $0.01. Participants can donate to their chosen charity from their endowments, but donations crowd out the experimenter’s donation $1:$1. Participants make their donation decisions while observing a neutral image on the computer screen, i.e., a grey circle. The image of a grey circle is included in the NoEyes control treatment to allow us to draw causal conclusions from changes in the treatment variable. If no image were included in the NoEyes treatment, both the presence and type of image would have changed between the NoEyes and the other two treatments. In this treatment, self-image concerns are active but social image concerns should not be. Running sessions on MTurk means that participants are truly anonymous to the experimenter and could not be watched.

**Fig 1:**
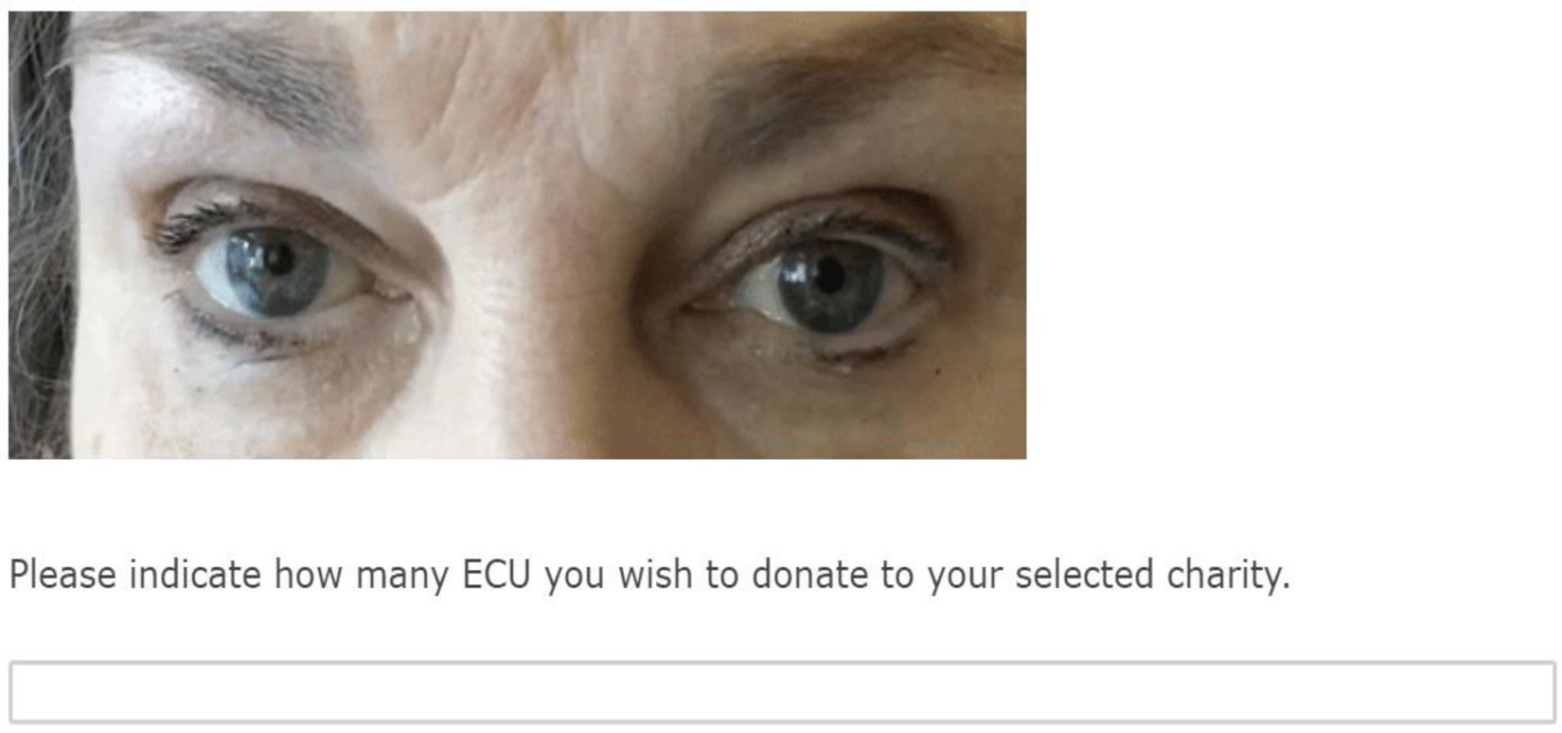
Screenshot of eyes from video.

*Treatment 2 (***DynamicEyes***)*: Same as treatment 1 but with a dynamic image (i.e., a 26-second video on a loop) onscreen of real eyes looking out at the participant and down at the keyboard while participants are making their donation decisions (a screenshot from the video is provided in Fig 1). No explanation for the eyes is provided, they are just there when the participant clicks onto the donation screen. By including the eyes on the screen, both self- and social image concerns are active. This enables us to capture the marginal impact of social image on warm-glow giving.

*Treatment 3 (***TurnOffEyes***)*: Same as treatment 2 but participants are introduced to the video and told the video would remain on while they make their donation decisions unless they choose, at no cost to themselves, to turn the video off. In this treatment, we capture an individual’s revealed preference for not having the watching eyes present while making their donation decision.

To determine whether our inclusion of the dynamic eyes had the effect we hypothesized, in a post-experiment survey, we asked participants in the **DynamicEyes** and **TurnOffEyes** treatments: “What did you think about the eyes?” Responses were varied and are discussed in Section 5.3: What did you think about the eyes? We also asked participants “Why did you choose to donate/not donate to your chosen charity?” Again, we received varying responses, and these are discussed in Section 5.2: Responses to Why did you choose to donate/not donate to your chosen charity? Question.

Based on our power analysis, our aim was to recruit approximately 300 participants in each treatment. The power analysis can be viewed in the preregistration document (Self- versus social image in warm-glow giving: https://osf.io/ej7d3/). We collected 1,423 responses. We eliminated duplicates and those who failed to answer the two comprehension check quiz questions correctly. These do not appear in our dataset. We ended up with a sample of 960 participants: **NoEyes**, *n* = 309; **DynamicEyes**, *n* = 314; **TurnOffEyes**, *n* = 337.

The data from the experiment is reported in S5 Appendix. The Monash University Human Research Ethics Committee approved the protocol for this study (Project ID: 25385).

## 4. Hypothesis

In this section, we provide our hypothesis along with the intuition behind our hypothesis. For a detailed derivation please refer to S2 Appendix.

In our setting donors face a trade-off between private consumption and both self- and social image; give more (less) and look better (worse) to one’s self and to others. The use of social norms has been shown to be effective in increasing charitable giving [57, 58]. Norms are important for both self- and social image. Societal norms help individuals determine if they should give and how much to give to maintain their desired self- and social image.

Prying eyes are less necessary for the individual to understand that giving is essential to maintain the desired self-image. However, without the fear of diminished social image, the amount given is less important. The individual can, via motivated reasoning [59–61] convince him/herself that the amount given, however meager, is the socially acceptable amount. Knowing, however, that prying eyes are watching, and judging, means the donor risks diminished social image if the donation is less than what the societal norm prescribes. The individual can still, via motivated reasoning, attempt to justify a meager donation, but it is no longer just the self that needs to be convinced, it is the others who observe the amount given.

We believe donors in the DynamicEyes and TurnoffEyes treatments will incur a greater marginal utility from giving than those in the NoEyes treatment, as they also receive utility from social image (from being “seen” to donate). Donors incur disutility from reduced income. Donors maximize utility by equating the marginal utilities derived from self-image and social image with the marginal disutility derived from reduced income.

It is possible that the watching eyes, if associated with the eyes of a potential recipient of any donation, may also strengthen self-image concerns. For example, if a participant associates the eyes with a recipient of the chosen charity and the eyes are interpreted as requesting help, this may trigger feelings of guilt. Thus, the watching eyes may have an effect through both further stimulating self-image as well as social image. While this may be possible, the impact of the eyes on self-image is likely to be minimal given our design. In our design, nothing is said about the person behind the eyes. Moreover, participants already know that all moneys will be going to a charity, one that they selected themselves from a list of ten; a selection they made before seeing the eyes. They would have no reason to assume that the eyes were related to a client of their chosen charity. Furthermore, even if the eyes are those of a recipient of a charity, the likelihood that the eyes represent a client of the chosen charity would be quite low given the fact we include 10 different charities.

It is also possible that the eyes, if associated with a recipient of the chosen charity, enhance altruistic preferences, and encourage greater charitable giving. While possible, the $1:$1 crowding-out feature of our design would discourage an altruist from giving as it would not benefit the charity’s recipients. An altruist, wishing to benefit the recipients, would be inclined not to give in the experiment but, instead, donate from their earnings afterwards.

If giving is positive when donors do not face social image concerns, then it will also be positive when they do. However, it is possible for giving to not be positive when donors do not face social image concerns, but positive when they do. Furthermore, when donors are concerned about both their self-images and their social images, they will donate more than when they are only concerned about their self-images. These points are summarized in the following hypothesis.

**Hypothesis**: Concerns over social image might positively affect giving at the intensive and extensive margin.

There are four types of individuals: Pure altruists, Warm-glow givers, Impure altruists, and Nongivers. We believe that social image concerns should have no effect on Pure altruists and Nongivers. For Warm-glow givers and Impure altruists there are those who are only concerned with self-image (type 1), those who are only concerned with social image (type 2), and those who are concerned with both (type 3). If there are enough of the second and third types, then giving at the intensive and extensive margin might increase when we activate social image concerns.

## 5. Results

Table 1 in S3 Appendix provides a summary of the socioeconomic characteristics for the participant pool across the three treatments. Successful randomization is indicated by the lack of significant differences across treatments, with the exceptions of two Big 5 measures (after applying the Bonferroni correction, only the difference pertaining to Conscientiousness remains significant).

### 5.1 Main results

**Result 1**: Activating social image concerns by creating the appearance of being watched does not significantly (after adjustment for multiple hypothesis testing) increase the likelihood of warm-glow giving. Participants donate at approximately the same rate across the three treatments.

**Fig 2:**
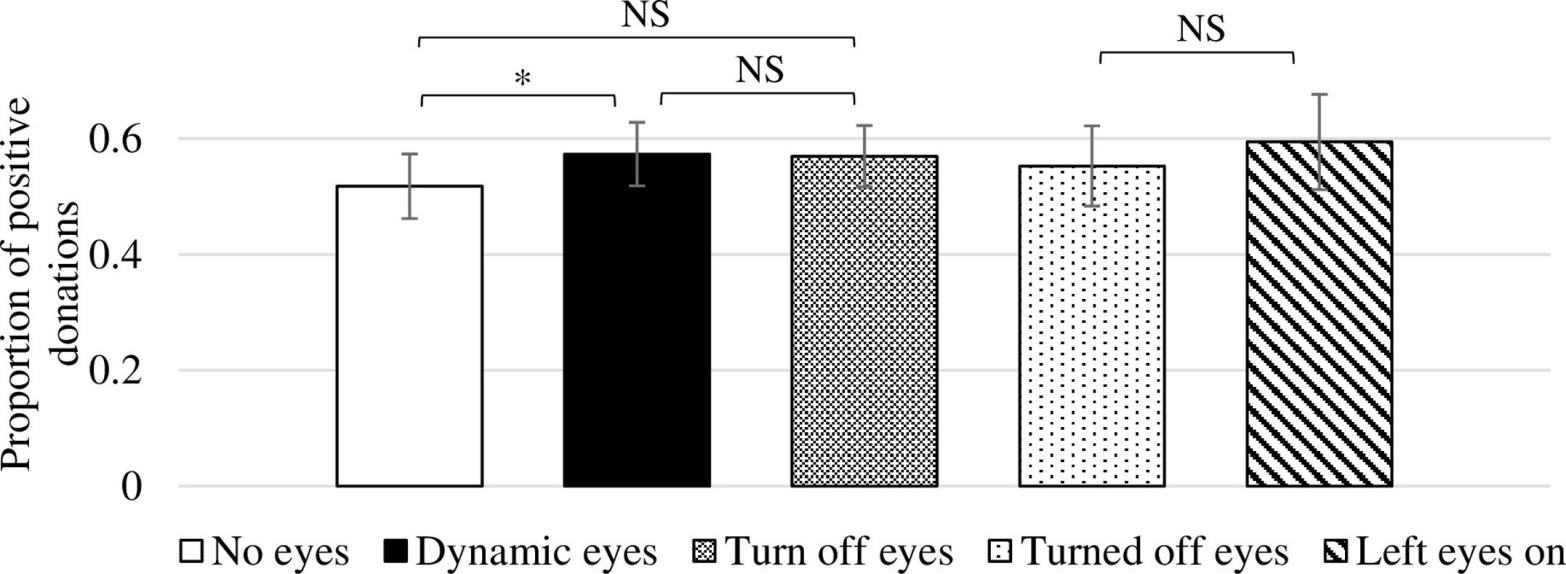
Proportion of positive donations across treatments. *Notes*: Error bars represent 95% confidence intervals.

Across the three treatments, between 50 and 60% of participants make a positive donation (**NoEyes**: 51.8%; **DynamicEyes**: 57.3%; **TurnOffEyes**: 57.0%–**TurnedOffEyes**: 55.3%; **LeftEyesOn**: 59.4%); see Fig 2, error bars represent 95% confidence intervals. Pairwise Fisher’s exact tests indicate no significant differences between some treatments: **NoEyes** vs. **TurnOffEyes**, *p*-value = 0.107 (all *p*-values reported are for one-tailed tests in accordance with our preregistration, unless otherwise indicated); **DynamicEyes** vs. **TurnOffEyes**, *p*-value = 0.496. However, we do see significant differences in the proportion that gave at the 10% level when comparing the **NoEyes** treatment to the **DynamicEyes** treatment: **NoEyes** vs. **DynamicEyes**, *p*-value = 0.095. After applying the Bonferroni multiple hypothesis test adjustment, none of the comparisons reveals a significant difference (a *p*-value < 0.035 is required for significance at < 0.10). A Kruskal-Wallis test for a difference across the three treatments also indicates no significant difference (two-tailed *p*-value = 0.295).

Our results replicate the rate of giving of both laboratory studies [14, 16, 18] and MTurk studies [19, 20]. In these studies, social image concerns were minimal and between 40 and 60% of participants donated.

Result 1 is confirmed by a logit regression of Donate (= 1 if donation > 0) on dummy variables for the **DynamicEyes** and **TurnOffEyes** treatments (Table 2 in S3 Appendix); **NoEyes** is the control group. Marginal values for the two treatment dummies equal 0.055 and 0.051, respectively, and they are both insignificantly different from zero and from one another (two-tailed *p*-value = 0.928).

**Result 2**: Creating the appearance of being watched significantly increases warm-glow giving at the intensive margin, but self-image accounts for the majority of all giving.

**Fig 3:**
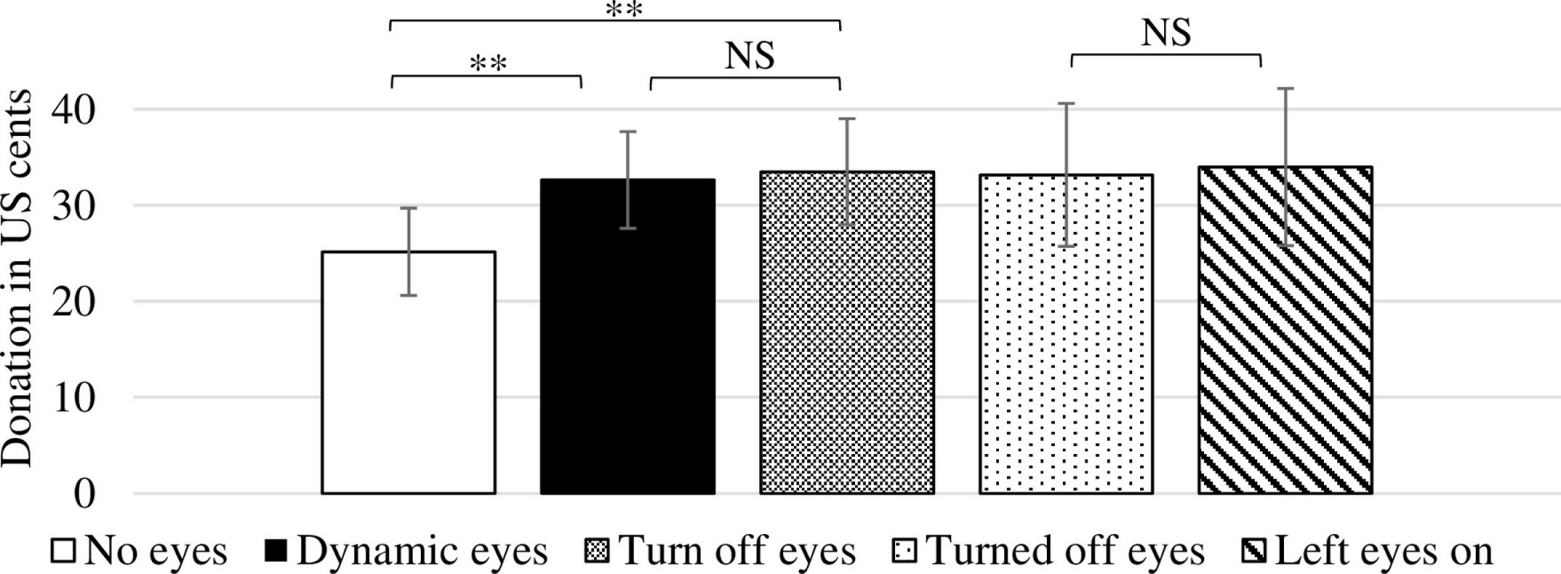
Average donations across treatments. *Notes*: Error bars represent 95% confidence intervals.

The average donation motivated by self-image, in the **NoEyes** treatment, is $0.25, approximately 12.5% of the endowment (see Fig 3). Activating social image with the watching eyes increases the average donation by approximately 33%. The average donation in the **DynamicEyes** (**TurnOffEyes)** treatment equals $0.33 ($0.34). Pairwise t-tests tests indicate significantly higher giving in the two Eyes treatments than the **NoEyes** treatment: **NoEyes** vs. **DynamicEyes** (*p*-value = 0.016); **NoEyes** vs. **TurnOffEyes** (*p*-value = 0.012); and **NoEyes** vs. combined **DynamicEyes** and **TurnOffEyes** treatments (*p*-value = 0.007). Applying the Bonferroni multiple hypothesis test adjustment, a *p*-value < 0.017 is required for significance at < 0.05. A Kruskal-Wallis test for a difference across the three treatments indicates a significant difference (*p*-value = 0.055).

Result 2 is confirmed by both a Tobit regression (with censoring at 0 and 200) and an OLS regression of Donation (= amount passed to the charity) on the **DynamicEyes** and **TurnOffEyes** dummy variables with **NoEyes** is our control group (Table 3 in S3 Appendix).

**Result 3:** A majority (59%) of participants in the **TurnOffEyes** treatment turned off the eyes, but this did not alter their likelihood of donating, or the amount donated.

As indicated in Fig 3, there was no difference in the proportion of positive donations in the **TurnOffEyes** treatment between the 199 participants who turned off the eyes (**TurnedOffEyes**) and the 138 participants who left the eyes on (**LeftEyesOn)**, 55.3% vs. 59.4% (*p*-value = 0.260). Likewise, as indicated in Fig 3, leaving the eyes on did not significantly alter average giving: **TurnedOffEyes**: $0.33; **LeftEyesOn**: $0.34; (*p*-value = 0.444).

Result 3 is confirmed by a logit regression, restricted to the **TurnOffEyes** subsample, of Donate on a dummy variable **LeftEyesOn** (= 1 if participant did not turn off the eyes) and a Tobit regression, restricted to the **TurnOffEyes** subsample, of Donation on **LeftEyesOn**, with censoring at 0 and 200 (Table 4 in S3 Appendix, columns 1 and 2, respectively).

### 5.2 Responses to why did you choose to donate/not donate to your chosen charity? Question

Participants in all treatments were asked in the post-experiment survey “Why did you choose to donate/not donate to your chosen charity?” We employed a textual analysis methodology to define categories and then to sort comments into these categories (see S4 Appendix for more details). This analysis was not preregistered. It is exploratory analysis. The program suggested a total of 10 categories: Financial Need/Personal Gain (*n* = 140); Charity Ineffectiveness (*n* = 192); Altruistic Reasons/Support for Charity (*n* = 199); Personal Connection to Charity (*n* = 62); Maximizing Earnings (*n* = 56); Mixed Feelings/Compromise (*n* = 18); Trust Issues with Charities (*n* = 10); Comments Expressing Personal Values (*n* = 24); Children/Youth Support (*n* = 77); and Other Reasons (*n* = 218). In Table 5 in S3 Appendix, we report three randomly selected (verbatim) comments assigned to each category.

Comments were not uniquely classified; some comments were assigned to more than one category. Of the 960 comments, four were classified to three categories and another 28 were classified to two categories. We accepted the classifications or comments as reported by GPT-4, except for four comments that were categorized to the Other Reasons plus at least one other category. After visual inspection of the comments, we dropped the Other Reasons categorization, allowing us to make pairwise comparisons of Other Reasons with the other nine categories. Table 6 in S3 Appendix, reports the Fisher exact test and t-tests two-sided *p*-values, respectively comparing categories to Other.

For participants with comments classified into one of the following four categories: Financial Need/Personal Gain; Maximizing Earnings; Charity Ineffectiveness; and Trust Issues with Charities, regardless of if the comments were also classified into a second (or third) category, we label as “Selfish.” These comments could be the rationalisations of “reluctant altruists” for not donating or donating little [37–41]. As noted previously, reluctant altruists want to appear prosocial, without bearing the cost of being prosocial. Reluctant altruists use excuses not only to reduce giving but also to not give at all [48]. What stands out in Table 6 of S3 Appendix, is that participants with comments classified as Selfish are less likely to give (≤ 20% vs. ≥ 72%) and give less on average (≤ 8 tokens vs. ≥ 30 tokens) than participants in any other category (Nonselfish).

In Table 1, we report by treatment frequency of giving and mean donation for Selfish and Nonselfish participants. We compare the two group’s frequency of giving and mean donation. For both groups, there is no significant differences across treatments.

**Table 1:** Frequency of giving and mean donation, selfish vs. nonselfish, by treatment.

Treatment	NoEyes	
	Selfish^+^ (*n* = 137)	Nonselfish^++^ (*n* = 172)
% giving	10.2%	84.9%
Mean Donation	1.8 (0.74)	43.76 (3.53)
Treatment	DynamicEyes	
	Selfish^+^ (*n* = 109)	Nonselfish^++^ (*n* = 205)
% giving	6.4%	84.4%
Mean Donation	4.4 (2.18)	47.6 (3.32)
Treatment	TurnOffEyes	
	Selfish^+^ (*n* = 129)	Nonselfish^++^ (*n* = 208)
% giving	10.9%	85.6%
Mean Donation	3.8 (1.82)	51.9 (3.91)
	Across Treatment tests	
	Selfish^+^	Nonselfish^++^
% giving Chi-square test *p*-value	0.456	0.944
Mean Donation Kruskal-Wallis *p*-value	0.500	0.366

*Notes*:

+ Participants with a comment classified to one of the following four categories: Financial Need/Personal Gain; Charity Ineffectiveness; Maximizing Earnings; and Trust Issues with Charities, regardless of any other classification.

++ Comment only classified to one of the following six categories: Altruistic Reasons/Support for Charity; Personal Connection to Charity; Mixed Feelings/Compromise; Comments Expressing Personal Values; Children/Youth Support; Other Reasons.

### 5.3 What did you think about the eyes?

Participants in the DynamicEyes and TurnOffEyes treatments were asked in the post-experiment survey “What did you think about the eyes?” We employed a textual analysis methodology to define categories and then to sort comments into these categories (see S4 Appendix for more details). This analysis was not preregistered. It is exploratory analysis. The program suggested a total of six categories: Emotion and Feeling (*n* = 283), Eye Color Perception (*n* = 126), Purpose or Influence (*n* = 54), Observation and Description (*n* = 181), Indifference or Minimal Impact (*n* = 46), and Other or Unrelated (*n* = 94). The classifications are again less than perfect; some comments are assigned to more than one category. Of the 651 comments, 18 were classified to three categories and another 95 were classified to two categories. We have accepted the classifications or comments as reported by GPT-4. The comments classified to the Other or Unrelated category are not also cross classified to any other category. We use this category for comparison purposes. In Table 7 of S3 Appendix, we report three randomly selected (verbatim) comments assigned to each category.

Table 2 reports the frequency of giving by category, average giving by category and Fisher exact test and t-test *p*-values, respectively comparing categories to Other. Three observations stand out. First, participants who made comments that were classified to the Other category were both significantly more likely to give than participants classified to the Emotion, Indifference, and Description categories; categories that included any comment related to the eyes. Second, donations are, after adjusting for multiple hypothesis testing, significantly higher for those in the Other category than those in any of the rest of the categories. Finally, both frequency of giving and donations are significantly higher for those in the Other category than for those in the rest of the categories combined (proportions test two-tailed *p*-value = 0.006; t-test two-tailed *p*-value = 0.001).

**Table 2:** What did you think about the eyes? Comments category: Donation rate, and donation amount by category.

	Emotion (*n* = 283)	Eye Color (*n* = 126)	Indifference (*n* = 46)	Description (*n* = 181)	Influence (*n* = 54)	Other (*n* = 94)
% donating	51.9%	54.8%	37.0%	50.8%	51.9%	70.2%
Fisher Exact test *p* Other vs. Emotion Other vs. Eye Color Other vs. Indifference Other vs. Description Other vs. Influence	= 0.003 = 0.025 < 0.001 = 0.002 = 0.033					
Average donation (Std Err.)	29.5 (2.83)	28.2 (3.95)	16.7 (3.94)	29.4 (3.42)	27.5 (5.28)	47.9 (6.2)
t-test (two-tailed *p*) Other vs. Emotion Other vs. Eye Color Other vs. Indifference Other vs. Description Other vs. Influence	= 0.008 = 0.008 < 0.001 = 0.010 = 0.013					

*Notes*: Emotion and Feeling = Emotion, Eye Color Perception = Eye Color, Purpose or Influence = Influence, Observation and Description = Description, Indifference or Minimal Impact = Indifference, Other or Unrelated = Other. Bonferroni multiple hypothesis test adjusted *p*-values: < 0.01 (0.003), < 0.05 (0.017), < 0.10 (0.033).

We next consider if being watched by the eyes makes participants more selfish than participants who were not watched. We regress Donate and Amount donated onto Selfish (see definition in Section 5.2: Responses to Why did you choose to donate/not donate to your chosen charity? question above), Saweyes (DynamicEyes and TurnOffEyes combined), and their interaction (Table 3). While selfish participants are, in general, significantly less likely to donate and donate less, there is no significant difference between those who observed the eyes and those who did not. This suggests that our classifications, selfish and nonselfish, may be indicative of more persistent “giving types” [62] and not a function of being or not being watched by the eyes.

**Table 3:** Effect of seeing the eyes and sounding selfish on donating and amount donated.

	Donated^+^	Amount Donated^++^
	Marginal Effect (Std. Err.) *p-value*	Coefficient (Std. Err.) *p-value*
Saweyes	-0.001 (0.028) *0.097*	6.50 (5.752) *0.259*
Selfish	-0.432*** (0.034) *<0.001*	-121.00*** (10.542) *<0.001*
Selfish × Saweyes	-0.019 (0.049) *0.700*	-3.76 (12.632) *0.766*
Pseudo R^2^	0.448	0.077
N	960	

*Notes*:

+ Logit regressions; Dependent variable: Donate (= 1 if donation > 0, otherwise 0)

++ Tobit regression: dependent variable = Amount donated, censored at 0 and 200; Saw Eyes is combined DynamicEyes and TurnOffEyes; NoEyes is the omitted treatment; *** 1%, ** 5%, * 10% significance level, one-tailed test. Bonferroni multiple hypothesis test adjusted *p*-values: < 0.01 (0.003), < 0.05 (0.017), < 0.10 (0.033).

The coefficient estimates in Table 4 indicate that those who were older, Caucasian, less religious, and had lower incomes were less likely to give. The coefficient estimates also suggest that Caucasians and those who were less religious gave less. The finding that religious participants were more likely to give and gave more is particularly interesting. Religious people might feel as though they are always being watched by their ever-present deity. No matter which treatment these people are exposed to, they feel a strong obligation to donate. The major difference between the Eyes and NoEyes treatments is that Caucasians are less likely to donate and donate less in the NoEyes treatment.

**Table 4:** Regression results: Personal attributes.

	Donated^+^			Donation Amount^++^		
	Marginal Effect (Std. Err.) *p-value*			Coefficient (Std. Err.) *p-value*		
Variable	Full sample	Eyes treatments	Noeyes	Full sample	Eyes treatments	Noeyes
Age	-0.003** (0.001) *0.050*	-0.001 (0.002) *0.597*	-0.006** (0.002) *0.011*	-0.230 (0.255) *0.367*	-0.001 (0.319) *0.998*	-0.630 (0.419) *0.133*
Gender^a^	-0.025 (0.031) *0.420*	-0.028 (0.037) *0.459*	-0.021 (0.053) *0.687*	-2.049 (5.376) *0.703*	-3.324 (6.657) *0.618*	-0.434 (8.875) *0.961*
Caucasian^b^	-0.079** (0.035) *0.026*	-0.044 (0.042) *0.300*	-0.163** (0.065) *0.013*	-14.555** (6.123) *0.018*	-6.096 (7.419) *0.412*	-32.350*** (10.825) *0.003*
Strong religion^c^	-0.011 (0.047) *0.807*	-0.006 (0.056) *0.909*	-0.024 (0.083) *0.770*	-1.482 (7.769) *0.849*	-1.429 (.551) *0.881*	-2.994 (13.048) *0.819*
Weak religion^d^	-0.315*** (0.044) *<0.001*	-0.309*** (0.053) *<0.001*	-0.337*** (0.078) *<0.001*	-49.590*** (8.493) *<0.001*	-52.204*** (10.520) *<0.001*	-45.524*** (14.086) *0.001*
Income^e^	0.001*** (0.000) *0.002*	0.001** (0.000) *0.028*	0.001** (0.001) *0.043*	0.107* (0.057) *0.060*	0.097 (0.071) *0.173*	0.103 (0.092) *0.267*
Constant				35.475*** (13.106) *0.007*	25.557 (15.754) *0.105*	59.191** (23.594) *0.013*
Pseudo R^2^	0.102	0.094	0.131	0.014	0.013	0.019
N	949	643	306	949	643	306

*Notes*:

+ Logit regression: dependent variable = Donated = 1 if donation > 0

++ Tobit regression: dependent variable = Amount donated, censored at 0 and 200; *, **, *** significant at < 0.10, 0.05, 0.01; a = 1 if male, One participant who responded Non-binary/Gender Diverse, My Gender identity is not listed, or Prefer not to say was excluded; b = 1 if Caucasian; c = 1 if response to: “My religion is very important to me,” was agree or strongly agree; d = 1 if response to: “My religion is very important to me,” was disagree or strongly disagree, the omitted group were those who responded “Neutral”, 10 participant were excluded for not answering this question; e = 25 if average income per year was less than $49,999, 75 if average income per year was $50,00-$99,999, 125 if average income per year was $100,000-$149,999, 175 if average income per year was $150,000-$199,999, 225 if average income per year was $200,000-$249,999, and 275 if average income per year was more than $250,000; f excludes participants who responded “Prefer not to say” to religion question. Bonferroni multiple hypothesis test adjusted *p*-values: < 0.01 (0.002), < 0.05 (0.008), < 0.10 (0.017).

### 5.4 Big 5 personality analysis

Finally, we consider if the likelihood of donating and the amount donated differ by participants’ personality traits as measured by the Big 5 personality traits: Extroversion, Agreeableness, Conscientiousness, Neuroticism, and Openness. Again, we combine DynamicEyes and TurnOffEyes. Results are reported in Table 5.

**Table 5:** Regression results: Big 5 personality traits.

	Donated^+^			Donation Amount^++^		
	Marginal Effect (Std. Err.) *p-value*			Coefficient (Std. Err.) *p-value*		
Trait	Full sample	Eyes treatments	Noeyes	Full sample	Eyes treatments	Noeyes
Extroversion	0.062*** (0.007) *<0.001*	0.069*** (0.008) *<0.001*	0.047*** (0.013) *<0.001*	8.548*** (1.394) *<0.001*	9.500*** (1.734) *<0.001*	6.099*** (2.313) *0.009*
Agreeableness	0.010 (0.009) *0.255*	-0.002 (0.011) *0.823*	0.034** (0.015) *0.023*	1.501 (1.620) *0.354*	-0.331 (2.027) *0.870*	4.678* (2.662) *0.080*
Conscientiousness	-0.047*** (0.009) *<0.001*	-0.032*** (0.011) *0.005*	-0.076*** (0.015) *<0.001*	-7.527*** (1.689) *<0.001*	-5.594*** (2.129) *0.009*	-10.049*** (2.729) *<0.001*
Neuroticism	0.008 (0.008) *0.366*	0.012 (0.011) *0.250*	0.002 (0.014) *0.911*	2.046 (1.523) *0.179*	3.838** (1.947) *0.049*	-0.876 (2.377) *0.713*
Openness	-0.047*** (0.008) *<0.001*	-0.045*** (0.010) *<0.001*	-0.046*** (0.013) *<0.001*	-6.792*** (1.467) *<0.001*	-7.031*** (1.050) *<0.001*	-5.953*** (2.275) *0.009*
Constant				41.290* (22.526) 0.067	28.232 (28.214) *0.317*	58.293 (36.436) *0.111*
Pseudo R^2^ ***R***^***2***^	0.102	0.103	0.117	***0.014***	***0.014***	***0.017***
N	960	651	309	960	651	309

*Notes*:

+ Logit regression: dependent variable = Donated = 1 if donation > 0

++ Tobit regression: dependent variable = Amount donated, censored at 0 and 200. Bonferroni multiple hypothesis test adjusted *p*-values: < 0.01 (0.002), < 0.05 (0.010), < 0.10 (0.020).

The coefficient estimates in Table 5 indicate that extroverts were more likely to give and gave more, regardless of treatment. Extroverts are typically highly sociable and warm. Hence, it is unsurprising that those who scored high in this trait were more likely to give and gave more as they feel the urge to contribute in a positive way to society.

The coefficient estimates also suggest that those scoring high in conscientiousness and openness to experience were less likely to give and gave less, again regardless of treatment. Conscientiousness is associated with being careful and diligent, while openness to experience is associated with intellectual curiosity and challenging authority. Therefore, it makes sense that those who scored high in each of these traits were less likely to give and gave less; they might be more likely to understand that, should they wish to do an altruistic act, donating to a charity in the context of the experiment was not the way to do so.

## 6. Conclusion

Prior research suggests approximately 50% of individuals are warm-glow givers. Whether these individuals give to maintain their self-images or to maintain their social images is something that requires investigation. We conduct an online experiment on MTurk in an attempt to bridge this knowledge gap. Participants are assigned to one of three treatments: the standard game (NoEyes) with self-image influences but minimal to no social image influence (participants are not “watched” as they make their donation); the social image influence game (DynamicEyes): participants are “watched” by a pair of dynamic eyes–a recording of a pair of actual eyes on a 26-second video loop–as they make their donation decisions; and the social image influence choice game (TurnOffEyes): Participants have the option of turning off the watching eyes before making their donation decisions.

We find that approximately 50% of our participants make a donation even though any giving was crowded out $1:$1, regardless of treatment, replicating the results of both laboratory and MTurk experiments. We find that participants donated significantly more in the **DynamicEyes** and **TurnOffEyes** treatments than in the **NoEyes** treatment. This suggests that warm-glow giving, while partially motivated by social image, is primarily motivated by self-image. We also find that approximately 59% of participants in our study chose to turn off the eyes prior to making their donation decisions, suggesting that participants have an aversion to being monitored while making donations, though it does not significantly impact the probability of giving or the amount given.

The use of social norms has been shown to be effective in increasing charitable giving [57, 58]. Norms are important for both self- and social image. Societal norms help individuals determine if they should give and how much to give to maintain their desired self- and social image. They face a trade-off between private consumption and both self- and social image; give more (less) and look better (worse) to one’s self and to others. Prying eyes are less necessary for the individual to understand that giving is essential to maintain the desired self-image. However, without the fear of diminished social image, the amount given is less important. The individual can, via motivated reasoning [59–61] convince him/herself that the amount given, however meager, is the socially acceptable amount. Knowing, however, that prying eyes are watching, and judging, means the donor risks diminished social image if the donation is less than what the societal norm prescribes. The individual can still, via motivated reasoning, attempt to justify a meager donation, but it is no longer just the self that needs to be convinced, it is the others who observe the amount given.

Our results suggest that charities looking to increase their donor bases might effectively do so by focusing on the self-image concerns of potential donors, while charities looking to increase the amount donated by their existing donors might effectively do so by focusing on the social image concerns of the donors. The inclusion of dynamic eyes on the screen while participants make donation decisions is essentially a nudge. The prominence and impact of nudges has grown significantly in recent years [63–65]. Like other nudges, the dynamic eyes on the screen imposed a very low cost to both the policymaker and the donor, and indeed had a positive impact on prosocial behavior. Consequently, this policy instrument is simple to implement on a large scale and can yield positive behavior change.

Warm glow has been used to justify behavior in a variety of settings, e.g., public health, voter turnout, environmental policy, business, and philanthropy. By expanding our understanding of the determinants of warm-glow motivated behavior we can effect change in many policy-relevant domains. Our findings suggest that policymakers wishing to encourage warm-glow motivated behavior should focus on highlighting the self-image incentives. Stressing social cues, though productive, might prove to be relatively less effective.

## Supporting information

S1 Appendix Instructions. (DOCX)

S2 Appendix Theoretical framework. (DOCX)

S3 Appendix Supplemental tables. (DOCX)

S4 Appendix Textual analysis methodology. (PDF)

S5 Appendix Data file and explanation. (XLSX)
