# Supplementary material for: It’s not you (well, it is a bit you), it’s me: Self- versus social image in warm-glow giving
Source: PLoS One. 2024 Mar 25;19(3):e0300868. doi: 10.1371/journal.pone.0300868 (PMC10962791; doi:10.1371/journal.pone.0300868)
Supplement: S1 Appendix — (DOCX) [file pone.0300868.s001.docx]

**Not for publication**

**Appendix A: Experiment Instructions**

Instructions common to all treatments are in *italics*.

*Welcome*

*This HIT consists of three Parts in total and will take approximately 10 minutes to complete. You are asked to answer some questions and make some decisions.*

*You will receive****$2.00****for completing all three Parts.*

***Part 1:***

***Charity choice***

*You will be paired with a charity of your own choosing selected from ten different charities listed below. A short description of each charity is also provided.*

***American Cancer Society***

*Provides many services to cancer patients and their families such as information, medical equipment, transportation to treatment locations, and a support system.*

***American Red Cross***

*Offers blood donation information and services, disaster relief, many helpful educational classes, as well as HIV/AIDS support groups.*

***Big Brothers Big Sisters***

*Provides one-to-one mentoring for youth and children residing in a one parent family for the purpose of creating caring, confident and competent young adults.*

***COVID-19 Solidarity Response Fund – WHO***

*Donations support WHO's work to track and understand the spread of the virus; to ensure frontline workers get essential supplies; and to accelerate research and development of a vaccine and treatments.****Doctors Without Borders***

*Doctors and nurses volunteer to provide urgent medical care in some 70 countries to civilian victims of war and disaster regardless of race, religion, or politics.*

***Feed the Children***

*One of America's most effective charities providing food, clothing, medical care, education, and emergency relief to children in the United States and overseas since 1979.****Oxfam America***

*Invests privately raised funds and technical expertise in local organizations around the world that hold promise in their efforts to help poor move out of poverty; committed to long term relationships in search of lasting solutions to hunger, poverty and social inequities.*

***Safe Horizon***

*Provides free and confidential services to survivors of intimate partner violence.****Sierra Club***

*Protects and preserves environmentally sensitive areas.*

***YMCA*** *Provides parent visitation monitoring services and physical fitness services.*

*Please indicate your charity of choice. Select one and only one charity.*

- *American Cancer Society ______*
- *American Red Cross ______*
- *Big Brothers Big Sisters ______*
- *COVID-19 Solidarity Response Fund – WHO ______*
- *Doctors Without Borders ______*
- *Feed the Children ______*
- *Oxfam America ______*
- *Safe Horizon ______*
- *Sierra Club ______*
- *YMCA ______*

*The charity you selected will receive $2.00 from the experimenter. From the $2.00 you have been paid, you have the option of donating to the charity you have just selected. You may donate as little or as much of your $2.00 as you wish.*

***PLEASE NOTE:****The amount contributed to your selected charity by the experimenter will be reduced by however much you pass to your selected charity. Your selected charity will receive neither more nor less than $2.00.*

***Before you make your donation decision, please answer the following question.***

*You elect to keep $1.50 for yourself and pass $0.50 to your charity of choice.*

*How much will you be paid for your participation today? (In USD)*

*How much will your selected charity receive? (In USD)*

**Noeyes**

​​​​​

Please indicate how much you wish to donate to your selected charity.

# **DynamicEyes**

#
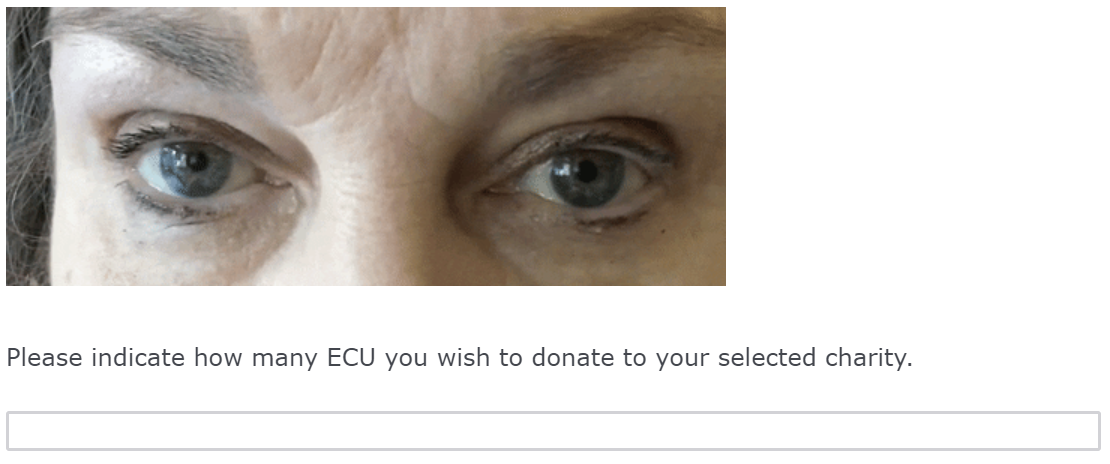


**TurnOffEyes**


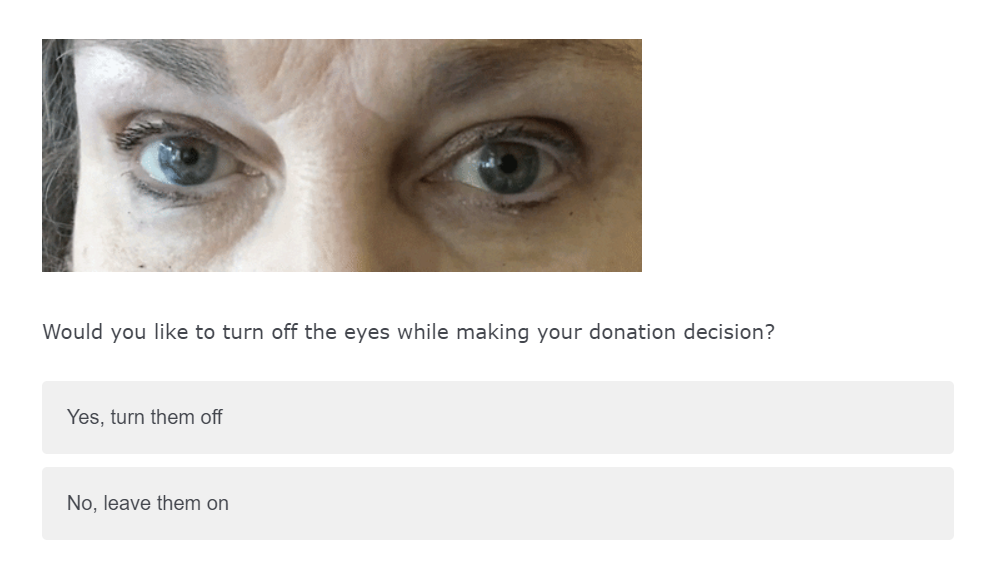


If participant left the eyes on:


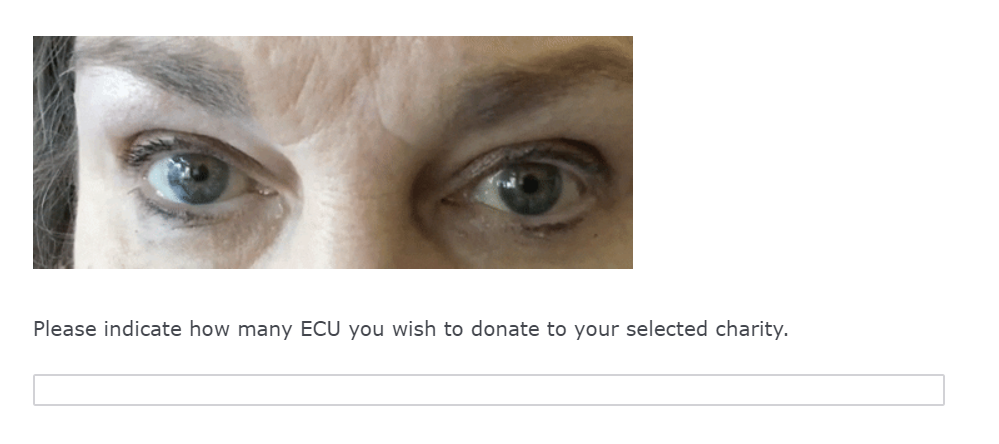


If participant turned the eyes off:


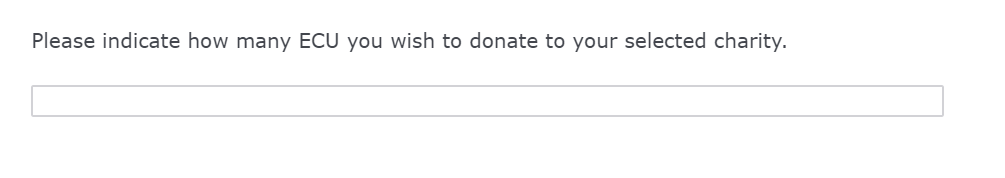


# ***Part 2****:*

# *Please answer the following demographic survey questions.*

*Age*

*Your Gender:*

*___Man*

*___Woman*

*___Non-binary/Gender Diverse*

*___My Gender identity isn’t listed*

*___Prefer not to say*

*Highest Level of Education:*

*___Not applicable*

*___Primary School*

*___High School*

*___College Undergraduate Degree*

*___Postgraduate Degree*

*Ethnicity:*

*___African American*

*___Caucasian*

*___Hispanic/Latino*

*___Asian*

*___Native American*

*___Other*

*___Prefer not to say*

*My religion is very important to me*

*___Strongly Disagree*

*___Disagree*

*___Neutral*

*___Agree*

*___Strongly Agree*

*___Prefer not to say*

*Average Income per year:*

*___Less than $49,999*

*___$50,00-$99,999*

*___$100,000-$149,999*

*___$150,000-$199,999*

*___$200,000-$249,999*

*___More than $250,000*

*In the past month, how many times have you volunteered your time to a charitable cause? ______*

*In the past month, how many times have you made a donation of money to a charitable cause? ____*

*[Chosen Charity] is a charity that supports a worthy cause?*

*___Strongly Disagree*

*___Disagree*

*___Neutral*

*___Agree*

*___Strongly Agree*

*“Think about the last time you gave to a charity before today. What was more important to you:”*

*___The total amount given by everyone, or*

*___The amount that you personally gave*

*___Both the total amount given by everyone and the amount you personally gave*

*___Some other aspect of giving*

DynamicEyes and TurnOffEyes

What did you think about the eyes?

*Why did you choose to donate/not donate to your chosen charity in Stage 1?*

*How many participants out of 10 do you believe donated to their charities of choice?*

# ***Part 3****:*

# *Please answer the following questions.*

***Instructions****: How well do the following statements describe your personality? Please place an X indicating how strongly you agree or disagree with each statement.*

*I see myself as someone who:*

|  |  | *Disagree Strongly* | *Disagree a little* | *Neither agree nor*  *disagree* | *Agree a little* | *Agree Strongly* |
| --- | --- | --- | --- | --- | --- | --- |
| *1* | *Is reserved* |  |  |  |  |  |
| *2* | *Is generally trusting* |  |  |  |  |  |
| *3* | *Tends to be lazy* |  |  |  |  |  |
| *4* | *Is relaxed, handles stress well* |  |  |  |  |  |
| *5* | *Has few artistic interests* |  |  |  |  |  |
| *6* | *Is outgoing, sociable* |  |  |  |  |  |
| *7* | *Tends to find fault with others* |  |  |  |  |  |
| *8* | *Does a thorough job* |  |  |  |  |  |
| *9* | *Gets nervous easily* |  |  |  |  |  |
| *10* | *Has an active imagination* |  |  |  |  |  |
