# Supplementary material for: It’s not you (well, it is a bit you), it’s me: Self- versus social image in warm-glow giving
Source: PLoS One. 2024 Mar 25;19(3):e0300868. doi: 10.1371/journal.pone.0300868 (PMC10962791; doi:10.1371/journal.pone.0300868)
Supplement: S2 Appendix — (DOCX) [file pone.0300868.s002.docx]

**Not for publication**

**Appendix B: Theoretical framework**

Donors have a vision of how prosocial they are, or they think they are, which we denote by $\varphi\in[0, 1]$. If $\varphi=0$, donors are not prosocial and do not care about how prosocial they appear to themselves. As $\varphi$ increases to 1, donors become more concerned about appearing to themselves as prosocial. Additionally, donors have a vision of how prosocial others see them, which we denote by $\varphi_{s}\in[0, 1]$. If $\varphi_{s}=0$, donors are not prosocial and do not care about how prosocial they appear to others. As $\varphi_{s}$ increases to 1, donors become more concerned about the opinion of others.

Donors hold beliefs about the giving of others which we denote by $\mu\geq0$. Donors wish to be at least equal to if not more prosocial than others. Donors control $\mu$; donors can through motivated reasoning (Kunda, 1990; Di Tella et al., 2015; Gino et al., 2016) direct $\mu$ towards 0; i.e., donors can convince themselves that others only give a small amount and therefore that is all that is required of themselves to maintain self-image. Hence, if $\varphi$ is sufficiently high, donors will give but they can convince themselves that the amount they give, however small, is sufficient. In the extreme, it becomes the act of giving, not the amount given, that matters.

Donors also hold beliefs about what others think is the appropriate amount to give, which we denote by $\mu_{s}\geq0$. These beliefs are only relevant when donors believe their actions are publicly observable. In this case, donors want to donate at least as much if not more than what others think is the appropriate amount. Donors cannot through motivated reasoning reduce $\mu_{s}$. It is a judgment of others that they have no control over.

The donor’s utility is defined as follows:

$$U=u_{1}\left( Y-g \right)+\varphi u_{2}\left( g-\mu\right)+\varphi_{s}fu_{3}(g-\mu_{s}),$$

in which,

$Y>0$ is the donor’s income (or endowment in our experiment), $g\geq0$ is the amount donated,$\mu, \mu_{s}, \varphi$ and $\varphi_{s}$are defined as above, and $f$ $\in\{0, 1\}$ is the degree of public observability. The first RHS term is utility from private consumption, the second term is utility from a prosocial self-image, and the third term is utility from a prosocial social image. We assume that $u_{1},u_{2}$and $u_{3}$ satisfy the conventional assumptions of monotonicity and concavity. Specifically, $u_{1}^{'}<0$, $u_{2}^{'}, u_{3}^{'}>0$ and ${u_{1}^{''}, u}_{2}^{''}, u_{3}^{''}<0$. Partially differentiating the donor’s utility function with respect to the amount donated yields

$$\frac{\partial U}{\partial g}=u_{1}^{'}+\varphi u_{2}^{'}+\varphi_{s}fu_{3}^{'}. (1)$$

There are two potential scenarios that we can explore in relation to equation (1). They are as follows.

**Case 1**: Let $f=0$, i.e., giving is not publicly observable. This scenario relates to the **NoEyes** treatment in our experiment.

Therefore, the optimal level of giving $g^{*}$, satisfies the following condition:

$$\frac{\partial U}{\partial g}=u_{1}^{'}+\varphi u_{2}^{'}=0,$$

$$\Rightarrow\varphi u_{2}^{'}={-u}_{1}^{'}.$$

Donors maximize utility by equating the marginal utility derived from self-image with the marginal disutility derived from reduced income.

**Case 2**: Let $f=1$, i.e., giving is publicly observable. This scenario relates to the **DynamicEyes** and **TurnOffEyes** treatments in our experiment.

Hence, the optimal level of giving $g^{**}$, satisfies the following condition:

$$\frac{\partial U}{\partial g}=u_{1}^{'}+{\varphi u}_{2}^{'}+\varphi_{s}u_{3}^{'}=0,$$

$$\Rightarrow\varphi u_{2}^{'}+\varphi_{s}u_{3}^{'}=-u_{1}^{'}.$$

Donors maximize utility by equating the sum of the marginal utilities derived from self-image and social image with the marginal disutility derived from reduced income.

We can illustrate the difference in the level of giving in cases 1 and 2 graphically, see Figure 1.

If giving is positive when it is not publicly observable ($f=0$), then it will also be positive when it is publicly observable, i.e., if $g^{*}>0$, then $g^{**}>0$. However, it is possible for giving to not be positive when it is not publicly observable but positive when it is publicly observable, i.e., $g^{*}=0$ and $g^{**}>0$. Furthermore, when donors are concerned about both their self-images and their social images, and $f=1$, they will donate more than when they are only concerned about their self-images i.e., $g^{*}<g^{**}.$ These points are summarized in the following hypothesis.

**Hypothesis**: Concerns over social image might positively affect giving at the intensive and extensive margin.

There are four types of individuals: Pure altruists, Warm-glow givers, Impure altruists, and nongivers. We believe that social image concerns should have no effect on Pure altruists and nongivers. For Warm-glow givers and Impure altruists there are those who are only concerned with self-image (type 1), those who are only concerned with social image (type 2), and those who are concerned with both (type 3). If there are enough of the second and third types, then giving at the intensive and extensive margin might increase when we activate social image concerns.


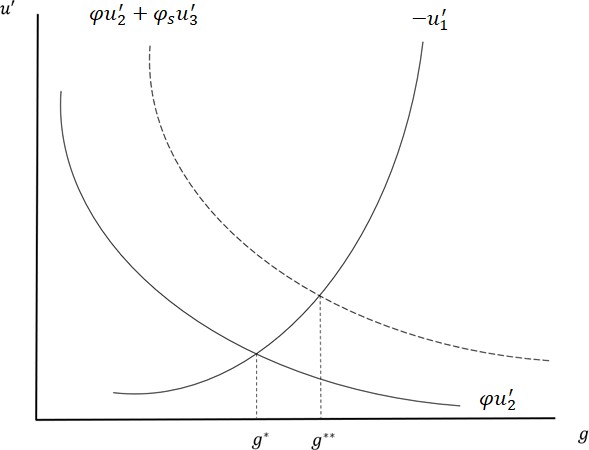


**Figure 1**: Optimal giving with and without public observation
