# Supplementary material for: It’s not you (well, it is a bit you), it’s me: Self- versus social image in warm-glow giving
Source: PLoS One. 2024 Mar 25;19(3):e0300868. doi: 10.1371/journal.pone.0300868 (PMC10962791; doi:10.1371/journal.pone.0300868)
Supplement: S3 Appendix — (DOCX) [file pone.0300868.s003.docx]

**Not for publication**

**Appendix C: Additional Materials**

**Table 1**: Summary statistics

|  | NoEyes | DynamicEyes | TurnOffEyes | Kruskal-Wallis test  *p*-value  (F-test *p*-value+) |
| --- | --- | --- | --- | --- |
| **Mean Age** | 37.60 | 36.51 | 35.86 | 0.13 |
| (Std. Err) | (0.62) | (0.60) | (0.56) | (0.11) |
| **Gender** |  |  |  |  |
| Man | 153 | 159 | 165 | 0.89* |
| Woman | 155 | 155 | 172 | (0.90) |
| Non-binary gender | 1 | 0 | 0 |  |
| **Education** |  |  |  |  |
| Not applicable | 0 | 0 | 1 |  |
| Primary School | 0 | 1 | 0 | 0.76 |
| High School | 86 | 70 | 72 | (0.82) |
| College Undergraduate Degree | 151 | 176 | 194 |  |
| Postgraduate Degree | 72 | 67 | 70 |  |
| **My religion is very** |  |  |  |  |
| **important to me** |  |  |  |  |
| Strongly disagree | 86 | 75 | 97 |  |
| Disagree | 35 | 28 | 29 | 0.37 |
| Neutral | 38 | 49 | 40 | (0.37) |
| Agree | 86 | 83 | 81 |  |
| Strongly agree | 62 | 78 | 83 |  |
| Prefer not to say | 2 | 1 | 7 |  |
| **Income** |  |  |  |  |
| < $49,999 | 140 | 142 | 141 |  |
| $50,00-$99,999  $100,000-$149,999  $150,000-$199,999 | 126  24  11 | 131  29  10 | 133  28  16 | 0.73  (0.25) |
| $200,000-$249,999 | 6 | 2 | 7 |  |
| > $250,000 | 2 | 0 | 4 |  |
| **Ethnicity** |  |  |  |  |
| African American | 22 | 26 | 37 |  |
| Caucasian | 246 | 225 | 244 |  |
| Hispanic/Latino Asian  Native American | 10  15  13 | 25  17  19 | 20  17  12 | 0.26  (0.51) |
| Other | 2 | 2 | 5 |  |
| Prefer not to say | 1 | 0 | 2 |  |
| **Extraversion** | 5.16 | 5.38 | 5.42 | 0.27 |
| (Std. Err) | (0.19) | (0.12) | (0.12) | (0.24) |
| **Agreeableness** | 6.91 | 6.97 | 6.96 | 0.95 |
| (Std. Err) | (0.11) | (0.11) | (0.10) | (0.91) |
| **Conscientiousness** | 7.75 | 7.19 | 7.63 | 0.003^β^ |
| (Std. Err) | (0.10) | (0.11) | (0.09) | (0.0002)^β^ |
| **Neuroticism** | 5.47 | 5.74 | 5.38 | 0.05 |
| (Std. Err) | (0.13) | (0.12) | (0.11) | (0.09) |
| **Openenss** | 7.12 | 7.04 | 7.22 | 0.39 |
| (Std. Err) | (0.11) | (0.10) | (0.10) | (0.44) |
| **Observations** | 309 | 314 | 337 |  |

*Notes*: * - chi-square contingency table test 𝑝-value; + Regression (OLS) of characteristic on treatment dummy variables, $p$-value of F-statistic. - Statistically significant after applying the Bonferroni correction.

**Table 2**: Decision to donate

|  | Marginal Effect  (Std Err.)  $p$-value | |
| --- | --- | --- |
| DynamicEyes | 0.055  (0.040)  *0.082* |  |
| TurnOffEyes | 0.051  (0.039)  *0.092* |  |
| Saw Eyes |  | 0.053  (0.034)  *0.059* |
| DynamicEyes=TurnOffEyes  two-tailed $p$-value | 0.928 |  |
| Pseudo R^2^ | 0.002 | 0.002 |
| N | 960 | |

*Notes*: Logit regressions; Dependent variable: Donate (= 1 if donation > 0, otherwise 0); Saw Eyes is combined DynamicEyes and TurnOffEyes; NoEyes is the omitted treatment; *** 1%, ** 5%, * 10% significance level, one-tailed test. Bonferroni multiple hypothesis test adjusted $p$-values: < 0.01 (0.003), <0.05 (0.017), 0.10 (0.033).

**Table 3**: Amount donated

|  | Tobit | | OLS | |
| --- | --- | --- | --- | --- |
|  | Coefficient  (Std Err.)  $p$*-*value | | | |
| DynamicEyes | 12.84*  (6.69)  *0.027* |  | 7.48**  (3.72)  *0.023* |  |
| TurnOffEyes | 14.25**  (6.58)  *0.016* |  | 8.33**  (3.65)  *0.012* |  |
| Saw Eyes |  | 13.57**  (5.792)  *0.010* |  | 7.919**  (3.202)  *0.007* |
| Constant | -4.42  (4.950) | -4.42  (4.950) | 25.17  (2.64) |  |
|  |  |  |  |  |
| Pseudo R^2^  R^2^ | 0.001 | 0.002 | 0.004 |  |
| Test  DynamicEyes=TurnOffEyes  two-tailed p-value | 0.827 |  | 0.814 |  |
| N | 960 | | | |

*Notes*: Dependent variable: Donation (= amount donated); Tobit regressions: censored at 0 and 200; Saw Eyes is combined DynamicEyes and TurnOffEyes; NoEyes is the omitted treatment; *** 1%, ** 5%, * 10% significance level, one-tailed test. Bonferroni multiple hypothesis test adjusted $p$-values: < 0.01 (0.003), <0.05 (0.017), 0.10 (0.033).

**Table 4**: TurnOffEyes Only: Decision to give and amount given

|  | 1 | 2 |
| --- | --- | --- |
|  | Logit+ | Tobit++ |
|  | Marginal Effect (Std Err.) | Coefficient  (Std Err.) |
| LeftEyesOn | 0.04  (0.055)  *0.225* | 3.84  (10.202)  *0.354* |
| Constant |  | 4.98  (6.860)  *0.234* |
| Pseudo R^2^  R2 | 0.001 | 0.0001 |
| N | 337 | |

*Notes*: TurnOffEyes treatment only; + Dependent variable: Donate (= 1 if donation > 0, otherwise 0); ++ Dependent variable: Donation (= amount donated); censored at 0 and 200; # Dependent variable: Ratio = (= Donation/average donation in the NoEyes treatment); TurnedOffEyes is the omitted variable; *** 1%, ** 5%, * 10% significance level, one-tailed test. Bonferroni multiple hypothesis test adjusted $p$-values: < 0.01 (0.003), <0.05 (0.017), 0.10 (0.033).

**Table 5**: “Why did you donate or not” comments categories: Three randomly selected comments by category, tokens donated, donation rate, and average donation amount

| **Comments (verbatim)** | **Tokens donated** | **% donating** | **Average donation**  **(Std Err)** |
| --- | --- | --- | --- |
| **Financial Need/Personal Gain (**$\boldsymbol{n=}$**140)** | | | |
| I don't work for free. Maybe if you'd actually paid a fair base wage. | 0 | 12.1% | 4.59  (1.75) |
| I do these type studies all the time where they ask you to donate, I am not sure sometimes if they will, and I always donate but at this time I am in bad need myself, thank you | 0 |  |  |
| Because either way they receive the 200 and I need the money but I also wanted to donate | 50 |  |  |
| **Charity Ineffectiveness (**$\boldsymbol{n=}$**192)** | | | |
| I chose not to donate because the charity would get the same amount, whether I donated or not. The charity wasn't impacted by my donation decision, only I was. I would have been affected negatively had I donated. | 0 | 4.7% | 0.7  (0.30) |
| My donation made no difference in the amount received, so I kept as much for myself as possible. I can always donate some of that amount so the charity benefits more in the end. | 0 |  |  |
| Because they would receive the same amount of money either way if I donated or not. | 0 |  |  |
| **Altruistic Reasons/Support for Charity (**$\boldsymbol{n=}$**199)** | | | |
| It's important to be charitable. | 10 | 95.0% | 48.0  (3.3) |
| To help others | 50 |  |  |
| I think it is a good cause | 1 |  |  |
| **Personal Connection to Charity (**$\boldsymbol{n=}$**62)** | | | |
| Because my mom had cancer | 1 | 75.8% | 31.5  (4.06) |
| I lost my son-in-law to cancer | 25 |  |  |
| I feel that we can survive anything but hunger and hunger makes you feel insignificant | 40 |  |  |
| **Maximizing Earnings (**$\boldsymbol{n=}$**56)** | | | |
| My donation made no difference in the amount received, so I kept as much for myself as possible. I can always donate some of that amount so the charity benefits more in the end. | 0 | 16.1% | 7.9  (4.25) |
| If I did NOT give to my chosen charity, then the charity and I MAXIMIZE our reward and that is the best outcome for everyone. If I wanted to, I could then turnaround and donate my $2.00 to my charity and they would get $4.00 instead of $2.00. | 0 |  |  |
| We each get the most money possible for the scenario that way. | 0 |  |  |
| **Mixed Feelings/Compromise (**$\boldsymbol{n=}$**18)** | | | |
| I do these type studies all the time where they ask you to donate, I am not sure sometimes if they will, and I always donate but at this time I am in bad need myself, thank you | 0 | 72.2% | 30.9  (9.51) |
| Because either way they receive the 200 and I need the money but I also wanted to donate | 50 |  |  |
| I wanted to help donate towards the cause but I also am living in tight financial constraints due to the pandemic so I had to find a compromise | 25 |  |  |
| **Trust Issues with Charities (**$\boldsymbol{n=}$**10)** | | | |
| Honestly, I don't trust the majority of charities. Too many of them don't give the majority of the funds to the cause and instead pay themselves ridiculous salaries and other overhead costs. | 0 | 20.0% | 4.0  (3.06) |
| the donation is helpful for other peoples, some charity is cheated for the peoples | 30 |  |  |
| I prefer to give on my own so I know it really goes there | 0 |  |  |
| **Comments Expressing Personal Values (**$\boldsymbol{n=}$**24)** | | | |
| I believe in the cause and think it is important to donate something as a gesture of meaning for me personally. | 50 | 95.8% | 53.8  (9.43) |
| Sierra Club does good work trying to protect the environment. | 0 |  |  |
| preservation of outdoor lands is important to me | 50 |  |  |
| **Children/Youth Support (**$\boldsymbol{n=}$**77)** | | | |
| because chids is the future of the nation. | 150 | 89.6% | 57.7  (6.57) |
| TO SUPPORT THE CHILDREN WHO ARE IN HUNGRY | 20 |  |  |
| I LIKE TO TAKE CARE OF CHILDREN AND WANT TO FEED THEM | 35 |  |  |
| **Other Reasons (**$\boldsymbol{n=}$**218)** | | | |
| Because I feel it. | 50 | 75.8% | 48.9  (3.78) |
| I just did not donate because they will still receive the 200 ECU regardless of what I pick. | 0 |  |  |
| Humanity | 50 |  |  |

**Table 6**: “Why did you donate or not?” comments category: Fisher Exact test and two-tailed t-test $p$-values

|  | % donating | Average donation |
| --- | --- | --- |
|  | Fisher Exact Test  $p$-value | Two-tailed t-test  $p$-value |
| Other vs. Fin need  Other vs. Charity ineff  Other vs. Max earnings  Other vs. Trust issues  Other vs. Altruistic  Other vs. Pers connect  Other vs. Mix Feel  Other vs. Pers values  Other vs. Children | <0.001  <0.001  <0.001  =0.006  <0.001  =1.00  =0.78  =0.02  =0.009 | <0.001  =0.002  <0.001  <0.001  0.86  =0.002  =0.09  0.63  0.25 |

*Notes*: Financial Need/Personal Gain = Fin need; Charity Ineffectiveness = Charity Ineff; Altruistic Reasons/Support for Charity + Altruistic; Personal Connection to Charity = Pers. Connect; Maximizing Earnings = Max earnings; Mixed Feelings/Compromise = Mixed feelings; Trust Issues with Charities = Trust issues; Comments Expressing Personal Values = Pers. Children/Youth Support = Children; Other Reasons = Other. Bonferroni multiple hypothesis test adjusted $p$-values: < 0.01 (0.001), <0.05 (0.006), 0.10 (0.011).

**Table 7**: Eyes comments categories: Three randomly selected comments by category, tokens donated, donation rate, and average donation amount

| **Comments (verbatim)** | **Tokens donated** | **% donating** | **Average donation**  **(Std Err)** |
| --- | --- | --- | --- |
| **Emotion and Feeling (**$\boldsymbol{n=}$**283)** | | | |
| I thought it was weird. | 0 | 51.9% | 29.5  (2.83) |
| alluring | 100 |  |  |
| they were freaky | 0 |  |  |
| **Eye Color Perception (**$\boldsymbol{n=}$**126)** | | | |
| They were a blue-grey and seemed out of place. | 1 | 54.8% | 28.2  (3.95) |
| SOMEWHAT BLUE | 2 |  |  |
| It is hard to tell the color of the eyes | 0 |  |  |
| **Purpose or Influence**  **(**$\boldsymbol{n=}$**54)** | | | |
| The eyes were somewhat influential in making my decision. | 40 | 51.9% | 27.5  (5.28) |
| At first, I was thinking of what color would best match them. When asked whether to keep or remove them, I knew what it was about. It didn't make a difference to me either way. | 0 |  |  |
| They made you feel like you were being watched and therefore judged about your choice | 50 |  |  |
| **Observation and Description**  **(**$\boldsymbol{n=}$**181)** | | | |
| those eyes were expecting something | 50 | 50.8% | 29.4  (3.42) |
| Interesting - needs better lighting to really see the color. | 0 |  |  |
| The eyes are normal | 20 |  |  |
| **Indifference or Minimal Impact** **(**$\boldsymbol{n=}$**46)** | | | |
| They didn't bother me. | 50 | 37.0% | 16.7  (3.94) |
| They were a bit weird to have but not a big deal | 15 |  |  |
| I don't feel anything. | 50 |  |  |
| **Other or Unrelated** **(**$\boldsymbol{n=}$**94)** | | | |
| i dont care this shit is so underpaid | 0 | 70.2% | 47.9  (6.2) |
| good | 150 |  |  |
| 2 | 50 |  |  |
