# Supplementary material for: It’s not you (well, it is a bit you), it’s me: Self- versus social image in warm-glow giving
Source: PLoS One. 2024 Mar 25;19(3):e0300868. doi: 10.1371/journal.pone.0300868 (PMC10962791; doi:10.1371/journal.pone.0300868)
Supplement: S4 Appendix — (PDF) [file pone.0300868.s004.pdf]

Appendix D

Ash and Hansen (2023) outline the use of text algorithms and Large Language Models (LLMs) such as GPT-4 in economics for tasks such as labeling text documents. Gilardi et al. (2023) show that GPT is better at annotation tasks than crowd-sourced workers, while Chiang and Lee (2023) discuss how annotations performed by an LLM can be more reliable and reproducible than humans.

In order to reliably implement LLMs into the annotation of the surveys conducted in this research, we draw on the 'Chain of Validation' introduced by Dhuliawala et al. (2023), who highlight the importance of critical deliberation over the final answer as a method of dealing with the potential for plausible yet incorrect responses termed 'hallucinations'. They also discuss that two alternative and complementary approaches are to use i) external tools or ii) using confidence scores. They suggest that as an individual method, chain of verification can be better than both of these alternative approaches.

Their suggested step-by-step process is to i) generate a baseline response ii) plan verification, iii) execute verification to test whether hallucinations exist, and iv) generate final verified response. They highlight that if the model is used to verify its own answer, extra care is needed to make sure it does not repeat all or part of its original mistake when cross-checking which facts are consistent.

To overcome this concern, we also draw on the methods highlighted by Cohen et al. (2023), who suggest using cross-examination of two language models in a multi-step process. The first language model generates a claim and the second model is asked a question to look for inconsistencies and conclude if the output is correct or incorrect, and to provide interpretable information to understand what mistakes are being made by the model and why.

To ensure interpretable and reliable output, we conduct the analysis in the following steps.

Step 1: Generating baseline response

In the first step, we generate a baseline response using Palm API from Google (Chowdhery et al. (2022); Anil et al. (2023)). To construct the prompt for this stage, we first create suggested categories for both groups of comments using ChatGPT. These categories are then used in the Palm query to utilize the few-shot capabilities of LLMs (Brown et al. (2020)).

We then construct a detailed prompt using a chain of thought methodology following Lightman et al. (2023), creating a detailed list of instructions to put each comment into one or more of the specified categories. The 'Palm Eyes Question Prompt' subsection shows an example of the final prompt. Using this prompt, we run a single survey response at a time.

Step 2: Planning verification

In the baseline response, the output is structured with the following columns which can be critically analysed by both human and another LLM to ensure accuracy:

1. **Category**: The category which the baseline results from the Palm API has been assigned as true.
2. **Applies**: A boolean column of whether or not Palm reasons that this category applies to the original comment.

3. **Phrases**: A direct citation of all or some of the original comment which the LLM used as evidence for the suggested category in that row.
4. **Original_Comment**: The original comment from the survey respondent.
5. **ID**: A unique ID for the original comment from the survey respondent.
6. **Category_Index**: An index number assigned to the category for both sets of question responses.

For the purpose of analysis, we include only rows where 'Applies' is true. Baseline reasoning given by the LLM of why this citation matches a given category makes the categorization interpretable.

To verify this information is correct, drawing on Cohen et al. (2023), in a second, critique-focused stage of the survey response labelling, the cited text of the comment, the original comment, and the reasoning from the baseline are analysed and critiqued by GPT-4. If both agree on the answer, then this category will be included in the final analysis. Subsections 'Dropped Eyes Responses' and 'Dropped Charity Response Comments' highlight responses that were dropped from analysis during data cleaning. For consistent structure of output, the prompts for both PALM and GPT-4 ask the LLMs to use a YAML format, which can be reliably processed into a data frame structure.

Step 3: Executing verification

Instead of reusing Palm and risking generating the same incorrect response (Dhuliawala et al. (2023)), we utilize the GPT-4 API (Brown et al. (2020); OpenAI, 2023) taking both the original input and the baseline output and performing a cross-examination of the suggested category, cited evidence, and provided reasoning. GPT-4 looks at the reasoning and evidence to see if it is i) backed up by the original comment and ii) agrees with the categories given by Palm. Subsections 'GPT-4 Verification Prompt Eyes Question' and 'GPT-4 Verification Prompt Charities Question' detail this process. We ask GPT-4 to output the following columns:

1. **GPT4_Applies**: A boolean column of whether GPT-4 agrees that an individual category in the baseline results applies to the 'Original Comment'.
2. **GPT4_Phrases**: A direct citation of all or some of the original comment which GPT-4 used as evidence of agreeing or disagreeing with the baseline suggested category in that row. In cases where GPT-4 does not agree with the baseline category, cells in this column will be false.
3. **GPT4_Reason**: The reasoning provided by GPT-4 for agreeing or disagreeing with the baseline category.
4. **GPT4_Incorrect_Reasoning**: A boolean, set to true if the baseline reasoning is not backed up by the original comment.

In order to reduce costs, we iterate through several baseline responses and use the PyYAML package to extract these into a structured data frame.

Step 4: Generating final verified response

Where both the baseline labelling by PALM and the follow-up labelling by GPT-4 are true, these are included for analysis. All other rows are dropped. The final dataset has reasoning from two LLMs, which can be interpreted by the reader for each individual

survey response. In cases where the reader disagrees with an individual category, this can be considered, and the analysis rerun with that specific change.

References

Anil, R., Dai, A. M., Firat, O., Johnson, M., Lepikhin, D., Passos, A., Shakeri, S., Taropa, E., Bailey, P., Chen, Z., et al. (2023). PaLM 2 Technical Report. https://arxiv.org/abs/2305.10403

Ash, E., & Hansen, S. (2023). Text algorithms in economics. *Annual Review of Economics*, *15*, 659–688.

Brown, T., Mann, B., Ryder, N., Subbiah, M., Kaplan, J. D., Dhariwal, P., et al. (2020). Language models are few-shot learners. *Advances in Neural Information Processing Systems*, *33*, 1877–1901.

Chiang, C. H., & Lee, H. Y. (2023). Can Large Language Models Be an Alternative to Human Evaluations? https://arxiv.org/abs/2305.01937

Chowdhery, A., Narang, S., Devlin, J., Bosma, M., Mishra, G., Roberts, A., et al. (2022). PaLM: Scaling language modeling with pathways. https://arxiv.org/abs/2204.02311

Cohen, R., Hamri, M., Geva, M., & Globerson, A. (2023). LM vs LM: Detecting Factual Errors via Cross Examination. https://arxiv.org/abs/2305.13281

Dhuliawala, S., Komeili, M., Xu, J., Raileanu, R., Li, X., Celikyilmaz, A., & Weston, J. (2023). Chain-of-verification reduces hallucination in large language models. https://arxiv.org/abs/2309.11495

Gilardi, F., Alizadeh, M., & Kubli, M. (2023). ChatGPT outperforms crowd-workers for text-annotation tasks. https://arxiv.org/abs/2303.15056

Lightman, H., Kosaraju, V., Burda, Y., Edwards, H., Baker, B., Lee, T., et al. (2023). Let's Verify Step by Step. https://arxiv.org/abs/2305.20050

Palm Eyes Question Prompt

```
Task: Analyze the given comment to categorize and explain it according
to the specified categories. When identifying keywords, consider full
phrases or sentence parts that directly relate to the category.
Input:
Comment: [{}]
Categories: ['Eye Color Perception', 'Emotion and Feeling',
'Purpose or Influence', 'Observation and Description',
'Indifference or Minimal Impact', 'Other or Unrelated']
Instructions:
1. Understand the overarching theme of the comment to guide
   subsequent steps.
2. Review the provided examples and descriptions for each
   category to familiarize yourself with the kind of phrases
   and sentiments associated with them.
3. Identify full phrases or sentence parts from the comment
   that directly relate to the categories.
4. Categorize the comment based on the identified phrases or
   sentence parts.
5. For each category, determine if it applies to the comment
   and provide a reasoning for the choice.
6. Organize your findings into the specified YAML output format.
7. Ensure accuracy of categorization and reasoning.
8. Reflect upon your analysis and ensure that you have captured
   all relevant sentiments from the comment.
9. Give an answer for the most likely category for each comment.
Descriptions and Examples:
1. **Eye Color Perception**:
   - Comments related to the perceived color of the eyes.
   - Examples: "blue", "grey"
2. **Emotion and Feeling**:
   - Comments expressing emotions or feelings evoked by the eyes.
   - Examples: "pretty", "creepy"
3. **Purpose or Influence**:
   - Comments speculating on the purpose or potential influence
     of the eyes in the survey.
   - Examples: "to guilt me into donating", "make me feel watched"
4. **Observation and Description**:
   - Comments describing the appearance or characteristics of the eyes.
   - Examples: "blinking", "odd"
5. **Indifference or Minimal Impact**:
   - Comments expressing indifference or stating that the eyes
     had minimal impact.
   - Examples: "didn't bother me", "didn't affect me"
6. **Other or Unrelated**:
   - Comments that don't fall into the above categories or provide
     unrelated information.
   - Examples: "N/A", "random"
Output Format (in YAML structure):
categories:
  - category: [Category Name]
    applies: [Boolean indicating if the category applies to the comment]
    reasoning: [Reasoning for the choice]
    phrases: [List of phrases or sentence parts from the comment
              that support the choice]
```

Figure 1: Eyes Prompt with Few-Shot Examples

Palm Charities Question Prompt

```
Task: Analyze the given comment to categorize and explain it
according to the specified categories. When identifying keywords,
consider full phrases or sentence parts that directly relate to
the category.
Input:
Comment: [{}]
Categories: ['Financial Need/Personal Gain',
'Charity Ineffectiveness', 'Altruistic Reasons/Support for Charity',
'Personal Connection to Charity', 'Maximizing Earnings',
'Mixed Feelings/Compromise', 'Trust Issues with Charities',
'Comments Expressing Personal Values', 'Children/Youth Support',
'Other Reasons']
Instructions:
1. Understand the overarching theme of the comment to guide
   subsequent steps.
2. Review the provided examples for each category to familiarize
   yourself with the kind of phrases and sentiments associated with them.
3. Identify full phrases or sentence parts from the comment that
   directly relate to the categories.
4. Categorize the comment based on the identified phrases or
   sentence parts.
5. For each category, determine if it applies to the comment and
   provide a reasoning for the choice.
6. Organize your findings into the specified JSON output format.
7. Ensure accuracy of categorization and reasoning.
8. Reflect upon your analysis and ensure that you have captured
   all relevant sentiments from the comment.
9. Give an answer for the most likely category for each comment.
Examples:
1. **Financial Need/Personal Gain**:
   - "I need the money."
   - "I want to keep as much money to myself as possible."
2. **Charity Ineffectiveness**:
   - "Donating wouldn't make a difference."
   - "No point in donating, charity gets the same."
3. **Altruistic Reasons/Support for Charity**:
   - "It's the right thing to do."
   - "I like to donate to charity."
4. **Personal Connection to Charity**:
   - "I am a cancer survivor."
   - "I lost my son-in-law to cancer."
5. **Maximizing Earnings**:
   - "I wanted to maximize my bonus."
   - "I wanted to keep the full 200."
6. **Mixed Feelings/Compromise**:
   - "I wanted to help but am living in tight financial constraints."
   - "I donated a small amount as a compromise."
7. **Trust Issues with Charities**:
   - "I don't trust the majority of charities."
   - "I worry about where the money goes."
8. **Comments Expressing Personal Values**:
   - "I believe in the cause."
   - "I believe in supporting worthy causes."
9. **Children/Youth Support**:
   - "I like to support children's charities."
   - "Children's lives are very important."
10. **Other Reasons**:
    - "Hurricane Harvey had just happened."
    - "Doctors dealing with people from all ethics is the greatest job for me."
Output Format (in YAML structure):
categories:
  - category: [Category Name]
    applies: [Boolean indicating if the category applies to the comment]
    reasoning: [Reasoning for the choice]
    phrases: [List of phrases or sentence parts from the comment
              that support the choice]
```

Figure 2: Palm Prompt with Examples for Comment Analysis

GPT-4 Verification prompt Eyes

```
base_prompt_with_examples = """
... [rest of your previous text] ...
"""

You are a helpful assistant. For each comment identified by an ID, check
if the given predicted categories apply based on the provided reasoning.
For each comment, provide the output in separate YAML documents, each
starting with '---' and structured as follows:
---
data:
  ID: (original ID)
  category_index: (e.g., c1, c2, ... corresponding to the original category_index)
  applies: (yes or no)
  phrases: (opinion if relevant phrases or sentence parts match original category)
  reason: (Reasoning why it does or does not match original category)
  incorrect_reasoning: (True or False original_comment does not match Reasoning)

ID: A1VOVSSAMR9R1C. Comment: 'I chose to donate out of my own pocket
because I wanted to feel like I was contributing to a cause and not just
letting an experimenter do the donation.' Reasoning: 'The comment
expresses a desire to contribute to a cause.' Category: 'Altruistic
Reasons/Support for Charity' Category_index: 'c3'
```

Figure 3: GPT-4 Verification Prompt with Examples and YAML Structure for Comment Analysis

```
You are a helpful assistant. For each comment identified by an ID, check
if the given predicted categories apply based on the provided reasoning.
For each comment, provide the output in separate YAML documents, each
starting with '---' and structured as follows:
---
data:
  ID: (original ID)
  category_index: (e.g., c1, c2, ... corresponding to the original category_index)
  applies: (yes or no)
  phrases: (opinion if relevant phrases or sentence parts match original category)
  reason: (Reasoning why it does or does not match original category)
  incorrect_reasoning: (True or False original_comment does not match Reasoning)

ID: A1JOGHF3FB1028. Comment: 'They didnt influence my decision as far as
I can tell. To be honest, they just kind of made me uncomfortable.'
Reasoning: 'The comment states that the eyes didnt influence the decision.'
Category: 'Indifference or Minimal Impact' Category_index: 'c5'
```

Figure 4: GPT-4 Verification Prompt with Examples and YAML Structure for Eyes Analysis

GPT-4 Verification prompt charities question

```
You are a helpful assistant. For each comment identified by an ID, check if the given
predicted categories apply based on the provided reasoning.
For each comment, provide the output in separate YAML documents, each starting with '---'
and structured as follows:
---
data:
  ID: (original ID)
  category_index: (e.g., c1, c2, ... corresponding to the original category_index)
  applies: (yes or no)
  phrases: (opinion if relevant phrases or sentence parts match original category )
  reason: (Reasoning why it does or does not match original category)
  incorrect_reasoning: (True or False original_comment does not match Reasoning)


ID: A1VOVSSAMR9R1C. Comment: 'I chose to donate out of my own pocket because I wanted to feel
like I was contributing to a cause and not just letting an experimenter do the donation.'
Reasoning: 'The comment expresses a desire to contribute to a cause.' Category: 'Altruistic
Reasons/Support for Charity' Category_index: 'c3'
```

Figure 5: GPT-4 Verification Prompt with Examples and YAML Structure for Charities Question Analysis

Example output

1. **category**: The category which the baseline results from the Palm API has been assigned as true.
2. **applies**: A boolean column of whether or not Palm reasons that this category applies to the original comment.
3. **Phrases**: This is a direct citation of all or some of the original comment which the LLM used as evidence for the suggested category in that row.
4. **original_comment**: This is the original comment from the survey respondent.
5. **reasoning**: This is the baseline reasoning given by the Palm2 API.
6. **ID**: This is a unique ID for the original comment from the survey respondent.
7. **category_index**: This is an index number assigned to the category for either of the input sets. This is used for reducing the monetary cost of output from GPT-4.
8. **gpt4_applies**: This is a boolean column of whether GPT-4 agrees with an individual category in the baseline results.
9. **gpt4_phrases**: This is the direct citation of all or some of the original comment which GPT-4 used as evidence of agreeing or disagreeing with the baseline suggested category in that row. In cases where GPT-4 does not agree with the baseline category, cells in this column will often be false.
10. **gpt4_reason**: The reasoning provided by GPT-4 for agreeing or disagreeing with the baseline category.
11. **gpt4_incorrect_reasoning**: A boolean, set to true if the baseline reasoning is not backed up by the original comment.
12. **incorrect_phrases**: Sometimes LLMs can give similar but different spelling for a small percentage of output. This is equivalent to gpt4_phrases. Will mostly be NULL.

```
---
- data:
    category: "Observation and Description"
    applies: TRUE
    reasoning: "The comment describes the appearance of the eyes."
    phrases: "['did not show much emotion']"
    original_comment: "The eyes looked gray in color, and did not
    show much emotion."
    ID: "A2KRWSCXNUNMAJ"
    category_index: "c4"
    gpt4_applies: TRUE
    gpt4_phrases: "The eyes looked gray in color, and did not show much emotion."
    gpt4_reason: "The comment describes the appearance of the eyes."
    gpt4_incorrect_reasoning: FALSE
    incorrect_phrases: NULL

---
- data:
    category: "Purpose or Influence"
    applies: TRUE
```

```
reasoning: "The comment speculates on the purpose of the eyes in the survey."
phrases: "['asking for a donation']"
original_comment: "Like they were asking for a donation"
ID: "A1ZD9SJXQ9C6EW"
category_index: "c3"
gpt4_applies: FALSE
gpt4_phrases: "Like they were asking for a donation"
gpt4_reason: "The comment does not speculate on the purpose of the eyes in the
survey.  Instead, it appears to associate a behavior or intent
(asking for a donation) with the eyes, which is metaphorical or figurative."
gpt4_incorrect_reasoning: TRUE
incorrect_phrases: NULL
```

Dropped eyes responses

[none, none, 10, na, na, 2, 2, none, na, 2, 2, 5, 2, 150, none, na, none, 12, na, 2, bold, na]

0.1 Dropped charity response comments

96 Responses are dropped in the charity question fall into the groups i) are nan or none, ii) are one word answers such as 'donate' or 'good' which are not easily categories. iii) any number cannot be reasonably sorted into a category iv) some comments such as 'ITS IS IMPORTANT FOR SAFE BORDERS' and 'I donate ALL THE TIME to causes and people. You guys guilt tripping pisses me off.' do not make it past the strict safety filter of the Palm API.

[50, 150, 50, 50, donate, 1, 150, 150, 150, Mturk, none, "I donate ALL THE TIME to causes and people. You guys guilt tripping pisses me off.", 150, 1, 150, 150, 150, 150, nothing, my opinion, 20, Money, DONATE, "im not donating shit this is paid shit already", 30, 10, 150, "ITS IS IMPORTANT FOR SAFE BORDERS", like, 1, nothing, no, interesting, good, good, good, 5, nothing, none, 150, 175, "TO SAVE PEOPLE FROM PARTNER VIOLENCE", 4, donate, "I am a survivor of domestic abuse", NOTHIG, Humanity, na, none, not, 200, na, Prayer, donate, 5, 150, 150, 150, 1, 2, 89, 25, 125, 160, 200, 200, donate, na, DONATE, 10, 150, 150, 10, 200, 150, EDUCATION, "I don't trust most charities, but I've never heard bad things about Doctors without Borders", donate, 150, 150, interesting, Good, 150, None, 150, na, Help, 150, 30, 1, DONATE, DONATE, 50, 150]
